# Application of Pharmacokinetic-Pharmacodynamic Modeling in Drug Delivery: Development and Challenges

**DOI:** 10.3389/fphar.2020.00997

**Published:** 2020-07-03

**Authors:** Huixi Zou, Parikshit Banerjee, Sharon Shui Yee Leung, Xiaoyu Yan

**Affiliations:** School of Pharmacy, Faculty of Medicine, The Chinese University of Hong Kong, Hong Kong, Hong Kong

**Keywords:** drug delivery, modified protein, pharmacokinetic modeling, pharmacodynamic modeling, mechanism-based PK-PD modeling, the minimal effective concentration

## Abstract

With the advancement of technology, drug delivery systems and molecules with more complex architecture are developed. As a result, the drug absorption and disposition processes after administration of these drug delivery systems and engineered molecules become exceedingly complex. As the pharmacokinetic and pharmacodynamic (PK-PD) modeling allows for the separation of the drug-, carrier- and pharmacological system-specific parameters, it has been widely used to improve understanding of the *in vivo* behavior of these complex delivery systems and help their development. In this review, we summarized the basic PK-PD modeling theory in drug delivery and demonstrated how it had been applied to help the development of new delivery systems and modified large molecules. The linkage between PK and PD was highlighted. In particular, we exemplified the application of PK-PD modeling in the development of extended-release formulations, liposomal drugs, modified proteins, and antibody-drug conjugates. Furthermore, the model-based simulation using primary PD models for direct and indirect PD responses was conducted to explain the assertion of hypothetical minimal effective concentration or threshold in the exposure-response relationship of many drugs and its misconception. The limitations and challenges of the mechanism-based PK-PD model were also discussed.

## Introduction

The research field of drug delivery focuses on the development of new techniques to manipulate drug absorption and disposition to achieve a desirable effect (Anselmo and Mitragotri, 2014; Asiri and Mohammad, 2018). With the advancement of technology, drug delivery systems with more complex architectures are developed. As a result, the drug absorption and disposition processes after administration of these drug delivery systems become exceedingly complex. The lack of understanding of the *in vivo* behavior of these delivery systems may limit their successful translation into clinics. Mechanism-based pharmacokinetic-pharmacodynamic (PK-PD) modeling could be used to untangle these complexities and improve the understanding of the *in vivo* behavior of these drug delivery systems, consequently informing their preclinical-to-clinical translation and clinical development.

**Figure 1:**
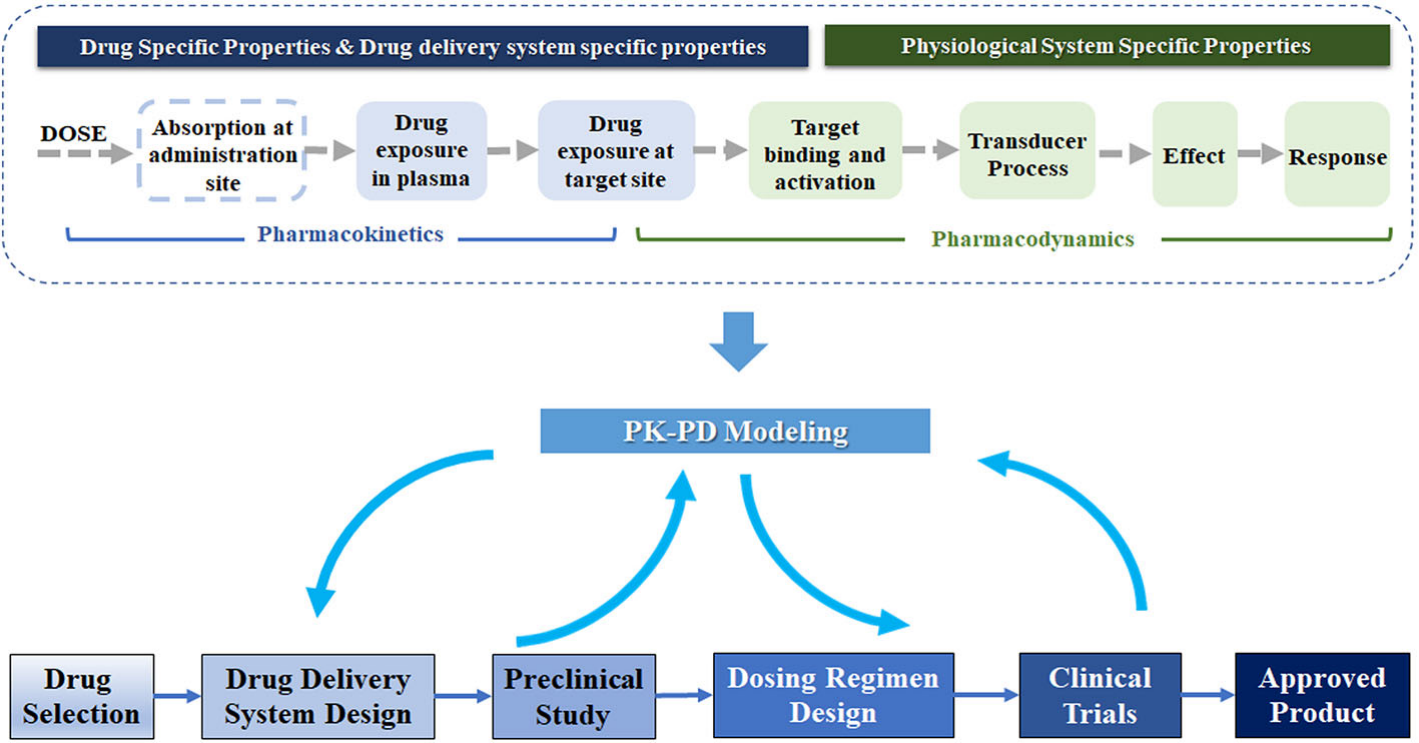
Schematic of PK-PD modeling in the drug delivery system development. In the development of the drug delivery system, PK-PD modeling could guide the formulation design and dosing regimen selection based on the preclinical and clinical data. This technique connects the drug dose to the physiological response, related to the properties of the drug delivery system and physiological system. A chain of events illustrates the flow from the administration, drug exposure (plasma and target site), receptor binding and activation, transduction to effect, and the effect on physiological response.

PK-PD modeling, an indispensable component of drug discovery and development, is a mathematical approach to study pharmacokinetics (PK), pharmacodynamics (PD), and their relationship (Peck et al., 1992; Danhof et al., 2005). As **Figure 1** shows, the mechanism-based PK-PD model can be incorporated into multiple stages in drug development. Explicitly, PK modeling quantitatively describes the process of absorption and disposition of drug in the body. PD modeling evaluates the time course of the pharmacological effects of drugs, with the consideration of the mechanism of drug action and major rate-limiting steps in the biology of the system (Mager et al., 2003). The PK and PD modeling can quantify the relationship of drug exposure and response, and further characterize the influences of drug-specific, delivery system-specific, physiological and pathological system-specific parameters on this relationship (Agoram et al., 2007; Danhof et al., 2007). Drug-specific parameters (e.g., drug clearance and receptor binding affinity) illustrate the interaction between the drug and the biological system. The drug delivery system-specific parameters represent the properties of carriers, such as the clearance, release rate, and the internalization rate of the carrier. The physiological system-specific parameters represent physiological values such as blood flow, life-span of cells, expression of enzymes, and transporters (Danhof et al., 2005; Danhof et al., 2007; Sager et al., 2015).

Through the separation of drug-specific and system-specific parameters in PK-PD modeling, the influences of various properties of the delivery system on the *in vivo* drug effect would be evaluated and facilitate its development. As shown in the bottom panel of **Figure 1**, the mechanism-based PK-PD models, developed based on the PK-PD data from preclinical studies, can be used to optimize the drug delivery system and predict the dosing regimen in humans. Once the clinical PK-PD data is available, they can be incorporated into the PK-PD models to further optimize their design. The PK-PD modeling can also evolve together with the clinical development to support the final approval.

Currently, modeling technique is commonly applied in the drug delivery system and modified large molecules. In the classic drug delivery system, modeling has been widely utilized in aiding the formulation design based on preclinical studies, such as liposome, nanoparticle, and nanoemulsion (Soininen et al., 2016; Benchimol et al., 2019; Kadakia et al., 2019). As for the modification of large molecules related to drug delivery, such as PEGylated protein, Fc-modified mAbs and antibody-drug conjugate (ADC), modeling technique has been widely used in both preclinical studies and clinical trials, providing valuable information for the animal-to-human translation and dose regimen selection in clinical trials (Mager et al., 2005; Zheng et al., 2011; Krzyzanski et al., 2013; Ait-Oudhia et al., 2017; McSweeney et al., 2018). There are also many review papers and book chapters on the recent advancement of modeling in drug delivery, while those publications focused more on pharmacokinetics (Yamashita and Hashida, 2013; Ait-Oudhia et al., 2014; Diao and Meibohm, 2015; Singh et al., 2015; Hedrich et al., 2018; Rodallec et al., 2018; Singh and Shah, 2018; Glassman and Muzykantov, 2019; He et al., 2019; Park, 2019). On the contrary, in this review, the linkage between PK and PD is highlighted. We introduce the basic theory of PK-PD modeling and its application relevant to drug delivery. The theory of PK focuses on the modeling of absorption and deconvolution, which is a technique used to identify an appropriate model structure for describing complex absorption. We further discuss the basic PD theory that links drug concentration and therapeutic effect. A few case studies, including the classic drug delivery system and modified large molecules, are presented to exemplify the application of this theoretical framework in practice. A model-based simulation is conducted to explain the assertion and misconception of a hypothetical minimal effective concentration or threshold, which has been used to guide the development of many drugs. Furthermore, the limitations and challenges of the mechanism-based PK-PD model are discussed.

## Basic Theory

### Pharmacokinetic Modeling

PK modeling is critical to understand the time courses of drug concentration following administration of various formulations and quantify the dose-concentration relationship. The method of compartmental modeling is commonly used to characterize PK (Jones and Rowland-Yeo, 2013). After the drug enters the central compartment (blood) *via* intravenous (IV) administration, distribution, and elimination occur. A one-compartment model is often used to describe the PK showing a monoexponential decline. It assumes the entire body (including blood, organs, and tissues) acts like a single, uniform compartment (Shargel et al., 1999). A two- or three-compartment model describes the PK curve that shows multi-exponential decay. Blood and well-perfused organs are usually lumped together and considered as a central compartment, while tissues with relatively slow but similar distribution rate are grouped together as one or more peripheral compartments (Ahmed, 2015). For drug administered *via* extravascular routes, its absorption to the central compartment is usually described by a first-order or zero-order process.

New drug delivery systems often significantly influence the PK by modifying the *in vivo* drug release and absorption process. By tuning the drug release profile, the apparent half-life of the drug could be prolonged, and drug accumulation at the target site may be enhanced (Shargel et al., 1999). Complex absorption can involve multiple pathways that occur simultaneously or sequentially, which could be challenging to model. Thus, modelers often use a numerical deconvolution technique to recover the complex drug absorption profile from the PK data so that an appropriate model structure can be used accordingly (Bonate and Steimer, 2011). More complex absorption models may consist of sequential and/or parallel combinations of the simple ones. Here, the simple model and basic techniques used in the absorption modeling are discussed, including first-order and zero-order absorption kinetics, flip-flop kinetics, and deconvolution. While other PK techniques, such as the population PK modeling and the physiologically based PK (PBPK) modeling, are also commonly used in the modeling of drug delivery system, basic concepts and principles of population PK and PBPK modeling are beyond the scope of this report. For readers who are interested with those two topics, several papers in the literature provided recent advancement and excellent review of these fields (Moss and Siccardi, 2014; Lestner et al., 2016; Li et al., 2017; Chetty et al., 2018; Nikanjam et al., 2018; Siccardi et al., 2018).

#### First-Order Absorption

**Figure 2:**
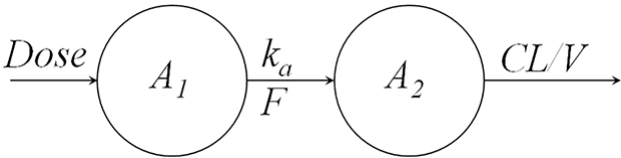
One compartment model with first-order absorption and first-order elimination.

The most common method to describe drug absorption after extravascular administration assumes a first-order absorption. **Figure 2** shows a one-compartment model with first-order absorption and first-order elimination. The model equations are as follows:

(1) $$\frac{dA_{1}}{dt}=-k_{a}\cdot A_{1}$$

(2) $$\frac{dA_{2}}{dt}=k_{a}\cdot A_{1}-\left(\frac{CL}{V}\right)\cdot A_{2}$$

(3) $$C_{p}=\frac{A_{2}}{V}$$

where *A_1_* denotes the mass of drug at the administration site, *k_a_* is the absorption rate, *A_2_* denotes the mass of drug in the body, *CL* represents the clearance, *V* represents the volume of distribution, and *C_p_* denotes the plasma drug concentration. The initial conditions for Eqs. 1 and 2 are:

(4) $$A_{1_{0}}=Dose\cdot F$$

(5) $$A_{2_{0}}=0$$

where *Dose* denotes the amount of drug administered, and *F* represents the bioavailability. By solving the above differentiation equations, the drug concentration, *C_p_*, can be expressed as:

(6) $$C_{p}=\frac{F\cdot k_{a}\cdot Dose}{V(k_{a}-\frac{CL}{V})}\cdot (e^{-\frac{CL}{V}\cdot t}-e^{-k_{a}\cdot t})$$

#### Zero-Order Absorption

Zero-order processes have also been used to describe the absorption after extravascular drug administration, where the drug is absorbed at a constant rate. The equations (Eqs.7 and 8) for the one-compartment model with zero-order absorption and linear elimination are:

(7) $$\frac{dA_{2}}{dt}=K_{0}-\left(\frac{CL}{V}\right)\cdot A_{2}$$

where *K_0_* represents zero-order input. The drug concentration in blood can be expressed as:

(8) $$C_{p}=\frac{K_{0}}{CL}\cdot \left(1-e^{-\frac{CL}{V}\cdot t}\right)$$

#### Flip-Flop Kinetics

**Figure 3:**
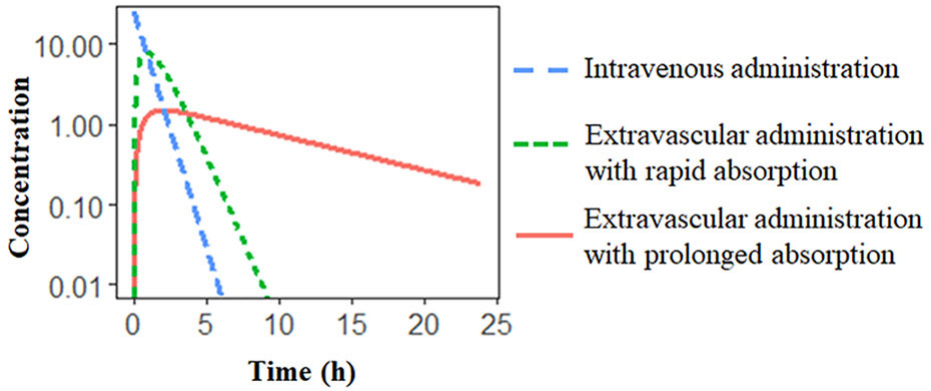
Schematic of extravascular administration with flip-flop kinetics. Simulated PK profile of the same drug to illustrate the effect of differences in the absorption rate. The slow absorption process results in a prolonged half-life.

A popular formulation approach is to extend the release of drug from the delivery system to reduce the dosing frequency and improve patient compliance (Stege et al., 1996; Jadhav et al., 2006). When the absorption process is much slower than the elimination process, the apparent half-life significantly increases due to the slow absorption step, resulting in flip-flop kinetics (Davis, 2018). For instance, in a one-compartment model with first-order absorption and elimination (**Figure 2**), when absorption rate *k_a_* is much smaller than the elimination rate *k_el_* (derived by *CL/V*), resulting in the flip-flop phenomenon. A schematic of flip-flop kinetics in **Figure 3** shows the simulated PK profile of a drug upon IV and extravascular administration. With a rapid absorption (*k_a_* > *k_el_*), the terminal slope of the concentration-time profile is similar to that after IV administration route, reflecting the *k_el_*. However, when drug absorption is slower than the elimination (*k*_a_ < *k*_el_), the absorption process becomes the rate-limiting step. The downward concentration-time curve is less steep and reflects the *k_a_*, while the upward curve reflects the elimination process, *k_el_*.

Flip-flop kinetics is commonly observed in the sustained- and controlled-release formulations (Idkaidek et al., 1998; Stepensky et al., 2001). However, the unawareness of flip-flop kinetics can lead to the incorrect characterization of the absorption process. In particular, the terminal phase of the PK profile might be controlled by drug absorption instead of the elimination, which cannot be distinguished with only PK data after extravascular administration. Therefore, the IV data is indispensable to recognize the flip-flop phenomenon and to accurately estimate the PK parameters associated with the drug absorption (Reed et al., 2017).

#### Deconvolution

Deconvolution has been widely used in PK modeling of drug absorption. It generates an input profile that can be used to guide the selection of the model structure for absorption (Deslandes et al., 1992). By deconvolution, one can estimate the rate and extent of absorption of various formulations *via* extravascular routes, such as subcutaneous, oral, intranasal, rectal, and transdermal (Larsen et al., 1991; Fiset et al., 1995; Björkman et al., 1997; Duquesnoy et al., 1998).

Deconvolution is achieved by the inverse operation of convolution, which is an approach to create a new function by combining two mathematical functions (Yanez et al., 2011). For example, the time courses of drug concentration in plasma after extravascular administration (Eq. 9) can be considered as a convolution of absorption and disposition functions and expressed as:

(9) F(t)=In(t)∗D(t)

where *F*(*t*) represents the function describing the drug concentration-time profile, and *In*(*t*) and *D*(*t*) denote the input/absorption function and output/disposition function, respectively. The symbol “*” represents the convolution operation. The disposition function *D*(*t*) can be obtained from the PK profile after IV administration. As long as PK profiles after IV and extravascular administrations are known, the profile for absorption function *In*(*t*) can be derived by numerical deconvolution algorithms in software. Currently, deconvolution algorithms are readily available in commercial software such as Phoenix WinNonlin 8.1 (Pharsight Corporation, Cary, North Carolina), Kinetica (Thermo Scientific), and GastroPlus (Simulations Plus, Lancaster, CA) (da Silva Honório et al., 2013; Balakrishnan, 2014).

### Pharmacodynamic Modeling

PD models quantify the relationship between drug concentration and therapeutic effect. In this section, the PD models that capture the main mechanisms of drug action are presented, including direct effect, biophase, and indirect response models. Similar to PK, PD is usually described by the compartmental models, and complex PD models are created by combining the simple model.

#### Direct Effect Models

At the beginning of the research field of pharmacodynamics, it is recognized that the pharmacological effect is linearly related to drug concentration or logarithm of drug concentration (Levy, 1964). It was supported by the clinical data of tubocurarine (Levy, 1966). The plasma drug concentration decreased exponentially after intramuscular injection, and the degree of muscle relaxation decreased linearly with time. However, the relationship between the effect and tubocurarine concentration is linear only if the effect is either less than 20% (linear) or within 20 to 80% of the maximum (log-linear) effect (*E_max_*) (Mager et al., 2003). Due to the limitation, the nonlinear E_max_ model was introduced (Wagner, 1968). The rationale for the E_max_ model is based on the classic receptor occupancy theory, and it assumes the drug effect (*E*) is directly proportional to the fraction of occupied receptors:

(10) E=γ⋅RC

where *RC* represents the concentration of drug-receptors complex, and *γ* is the proportional factor. The drug-receptor complex equilibrium function is described as below:

(11) $$RC=\frac{R_{0}\cdot C_{p}}{K_{D}+C_{p}}$$

where *R_0_* is the total receptor concentration in the tissue, and *K_D_* is the dissociation constant for the drug-receptor complex. By combining Eq. 10 to 11, the E_max_ model equation could be derived:

(12) $$E=\frac{E_{max}\cdot C_{p}}{EC_{50}+C_{p}}$$

where *E_max_* is the maximum possible effect and equal to *γ∙RC*, and *EC_50_* is a sensitivity parameter representing the drug concentration producing the half-maximal effect. The E_max_ model is frequently used to describe the *in vivo* exposure-effect relationship of many central system drugs and cardiovascular agents (Minematsu et al., 2001; Friberg et al., 2005), where a rapid onset of drug effect is induced. For instance, the E_max_ model has been used to describe the relationship between cocaine concentration and cardiovascular effect, including systolic and diastolic blood pressures as well as the heart rate (Laizure and Parker, 2009).

In addition to the linear relationship between the effect and the drug-receptor complex, a nonlinear relationship has also been proposed as the operational model of agonism (Black and Leff, 1983). The model is expressed as:

(13) $$E=\frac{E_{max}\cdot RC}{K_{E}+RC}$$

where *K_E_* is the concentration of the drug-receptor complex that triggers a half-maximum effect. By combining Eq.11 and Eq.13, the relationship between the drug effect and the concentration of agonist (A) can be derived as below:

(14) $$E=\frac{E_{max}\cdot \tau \cdot A}{K_{D}+(\tau +1)\cdot A}$$

where *τ* represents the operational efficacy of agonist and is defined by the total concentration of receptor (*R_0_*) divided by *K_E_*. It should be noted that the concentration of drug achieving maximum effect is no longer *EC_50_* as defined in Eq.12. As the concentration of drug (*A*) goes to infinity, the maximal effect is described by the asymptote parameter (*α*) of Eq.15:

(15) $$\alpha =\frac{E_{max}\cdot \tau }{1+\tau }$$

The concentration of agonist producing the half-maximal effect (*A_50_*) could be derived and shown in Eq. 16:

(16) $$A_{50}=\frac{K_{D}}{1+\tau }$$

In Eq.15, when *τ* is large ($\frac{\tau }{1+\tau }$  approaches to 1), *α* approaches to *E_max_*, and *A_50_* is much smaller than *K_D_*, suggesting the drug is a full agonist. However, when *τ* is small ($\frac{\tau }{1+\tau }$ approaches to 0), *α* is smaller than E_max_, and *A_50_* approaches to *K_D_*, indicating a partial agonism. The operational model of agonism suggested that the drug triggering effect is a two-step process, where the first step is the receptor binding process, and the second step is the signal-transduction process. Therefore, it can simultaneously analyze concentration-response data of compounds with different binding affinities (*K_D_*) and efficacy (*τ*), such as full and partial receptor agonists (Danhof et al., 2007). It has been applied in μ opioid (MOP) receptor agonists with varying affinities of receptor and intrinsic efficacies (Scott et al., 1991; Cox et al., 1999).

The models mentioned above (the E_max_ model and the operational model of agonism) describe the direct effect of drugs, when there is no time delay between plasma concentration and response. However, the lag between therapeutic effect and plasma concentration is commonly observed. Depending on the mechanism of the delay, this phenomenon can often be explained by either the biophase model or the indirect response model.

#### Biophase Distribution Model

**Figure 4:**
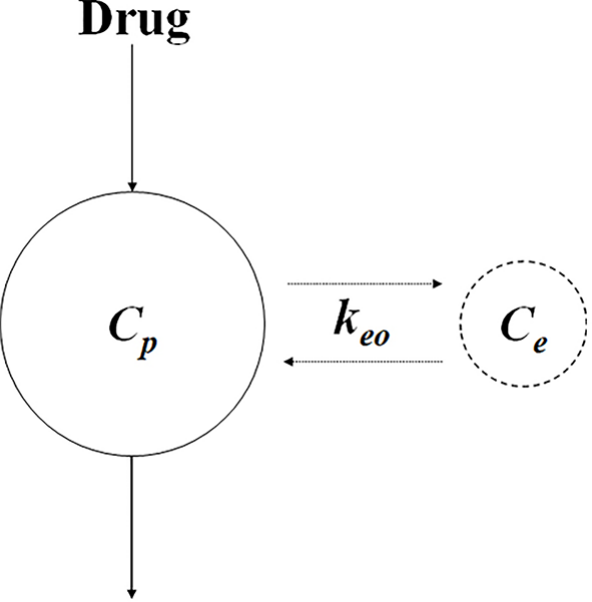
Schematic of biophase model. *C*_e_ and *C*_p_ represent the concentration at the biophase and plasma, respectively. *k*_eo_ represents the first-order distribution rate constant.

The biophase model attributes the delay between the drug concentration and therapeutic effect to the time that it takes for the drug in the plasma to distribute to the target site (Sheiner et al., 1979; Danhof et al., 2007). A biophase compartment was proposed to represents the drug at the target site. In **Figure 4**, a biophase model has been utilized to explain the delayed effect in relation to the plasma drug concentration of d-tubocurarine (Sheiner et al., 1979), where the plasma concentration is linked to biophase concentration with the following differential equation:

(17) $$\frac{dC_{e}}{dt}=k_{eo}\cdot C_{p}-k_{eo}\cdot C_{e}$$

where *C*_e_ and *C*_p_ represent the concentrations in the biophase and plasma, respectively, and *k*_eo_ represents the first-order distribution rate constant. As the delay in response due to the distribution process, it could be affected by the physicochemical properties of the drug (e.g., molecule size), binding to plasma protein, and transporter expression (Danhof et al., 2007).

#### Indirect Response Models

**Figure 5:**
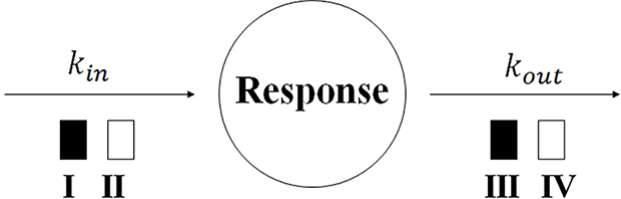
Indirect response model structure. *k_in_* and *k_out_* represent the first-order production constant and the first-order dissipation rate constant of the response, respectively. The open and solid box represents the inhibitory and stimulatory effect, respectively.

Indirect response (IDR) models are widely used to describe the delayed response generated by the indirect mechanism. Specifically, the drug could stimulate or inhibit either the production or the dissipation of drug response, causing the delay. Jusko and his group formalized four basic indirect response models to describe diverse clinical pharmacodynamic data (Dayneka et al., 1993; Jusko and Ko, 1994). As shown in **Figure 5**, Model I and Model II show the inhibitory effect on the input or loss of response, and Model III and Model IV show the stimulatory effect. The models are expressed as:

(18) $$\frac{dR}{dt}=k_{in}\left(1-\frac{I_{max}\cdot C_{p}}{IC_{50}+C_{p}}\right)-k_{out}\cdot R\;Model\;I$$

(19) $$\frac{dR}{dt}=k_{in}-k_{out}\left(1-\frac{I_{max}\cdot C_{p}}{IC_{50}+C_{p}}\right)\cdot R\;Model\;II$$

(20) $$\frac{dR}{dt}=k_{in}\left(1+\frac{S_{max}\cdot C_{p}}{SC_{50}+C_{p}}\right)-k_{out}\cdot R\;Model\;III$$

(21) $$\frac{dR}{dt}=k_{in}-k_{out}\left(1+\frac{S_{max}\cdot C_{p}}{SC_{50}+C_{p}}\right)\cdot R\;Model\;IV$$

where *R* represents the response; *I*_max_ and *S*_max_ are defined as the maximal effect of inhabitation or stimulation, respectively; IC_50_ and SC_50_ are the concentration trigger the half-maximal effect of inhibition or stimulation, respectively. The IDR model I has been applied to describe the PK-PD relationship of Warfarin, a vitamin K epoxide reductase inhibitor. Warfarin takes effect by inhibiting the production of prothrombin and cause a delayed anticoagulant effect (Nagashima et al., 1969). A number of papers have reviewed the applications of IDR models (Sharma and Jusko, 1998; Mager et al., 2003; Danhof et al., 2007). The IDR models can be extended to characterize more complex dynamics. A life-span based IDR model has been developed for therapeutic growth factors that alter the production of natural cells (Krzyzanski et al., 1999). A precursor-dependent IDR model has been used to describe the drug effect on the production of endogenous mediators from a specific precursor (Sharma et al., 1998).

Furthermore, integration of IDR model I with the operational model (Eq.22) has been applied in A_1_ adenosine receptor agonists with different binding affinities, to describe the relationship between their plasma concentrations and antilipolytic effects (Van der Graaf et al., 1997; Van der Graaf et al., 1999):

 (22) $$\frac{dR}{dt}=k_{in}\cdot \left(1-\frac{E_{max}\cdot \tau \cdot A}{K_{A}+(\tau +1)\cdot A}\right)-k_{out}\cdot R$$

The operational model with IDR model I (Eq. 22) might be preferred than the original IDR model I (Eq.18), because it assumes the nonlinear transduction between the drug-receptor complex and the effect of stimulation of *k_in_*. However, this model requires PK-PD data from both full and partial agonists to resolve the model parameters. Given such data are often not available, the original IDR model is more commonly applied to characterize the PK-PD relationship of a single compound.

### Application of PK-PD Modeling

In this section, we will discuss the application of PK-PD modeling in the development of four different types of drug delivery systems, including extended-release formulation, modified protein, liposome, and antibody-drug conjugates. Furthermore, a model-based simulation using a spectrum of basic PD models was conducted to illustrate the hypothetical minimum effective concentration (MEC).

**Figure 6:**
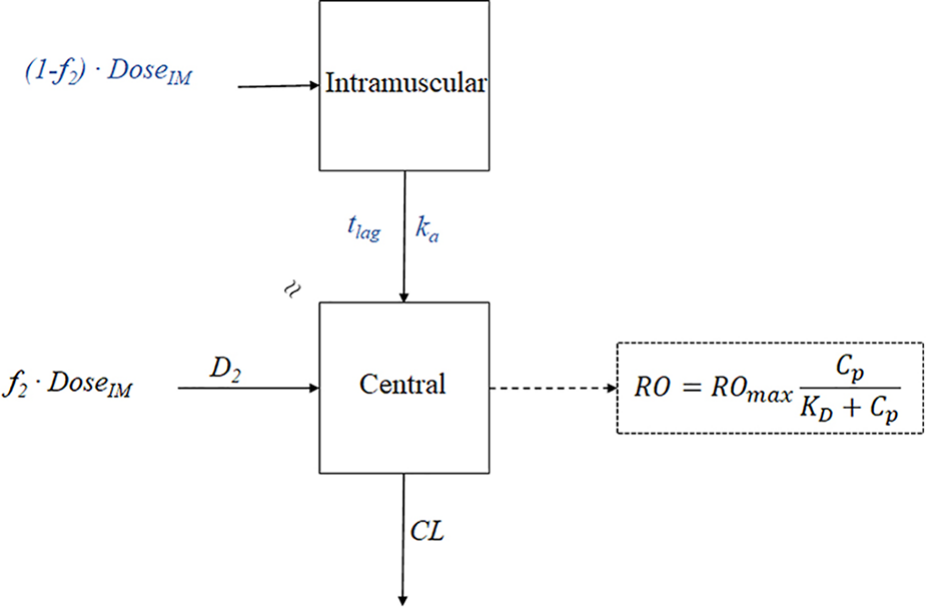
The PK-PD model of paliperidone palmitate. The absorption model was divided into two components: *f*_2_ and *(1- f_2_)*. *f_2_* denotes the fraction that enters the central compartment *via* a zero-order process, and *(1-f_2_)* denotes the fraction that enters the central compartment *via* a first-order process. *t*_lag_ is the lag time, *k_a_* is the absorption rate, *D_2_* is the duration of *f*_2_ for zero-process and equal to *t*_lag_, *CL* represents clearance, *RO* represents receptor occupancy, RO_max_ represents the maximal occupancy can be achieved, *C*_p_ represents the plasma concentration, and *K*_D_ is the equilibrium dissociation constant.

Complex PK-PD models, as shown in **Figures 6** **–** **9**, are often constructed by assembling basic PK and PD model components that have been discussed in previous sections. **Table 1** summarizes the building blocks of PK and PD models that are used to characterize the activity of the compounds in the case studies. For example, the PK-PD model for rHuEPO in **Figure 8** is a combination of the first-order absorption model, target-mediated drug disposition (TMDD) model (Mager and Jusko, 2001a), operational model of agonism (Black and Leff, 1983), precursor-pool dependent IDR model (Sharma et al., 1998), and transit compartment model for transduction process (Mager and Jusko, 2001b).

**Figure 7:**
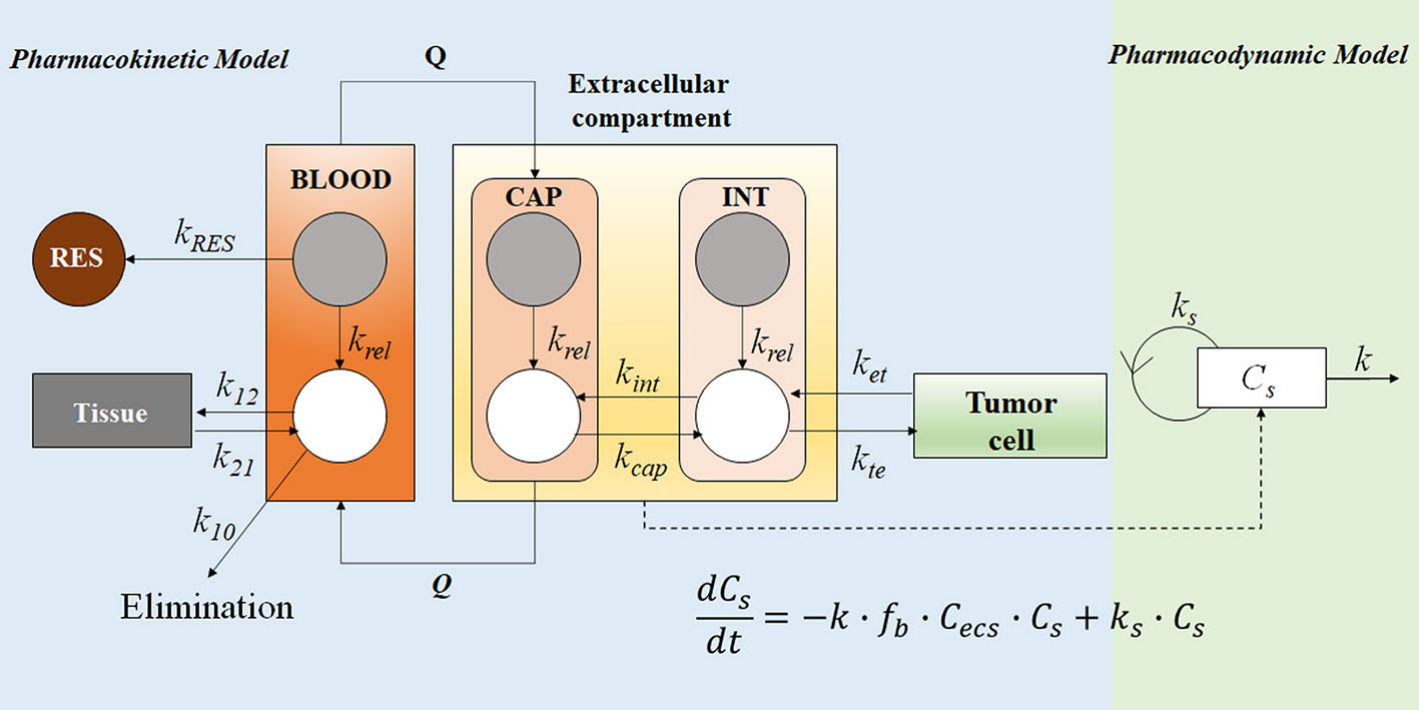
The PK-PD model of the free doxorubicin and liposomal doxorubicin. Free doxorubicin and liposomal doxorubicin are represented by white and grey circles, respectively; the disposition of free doxorubicin and liposomal doxorubicin was described by a two-compartment and a one-compartment model, respectively. The intra-tumor disposition is described by a physiological model linked with tumor blood flow rate (*Q*). Tumor tissue was divided into three compartments: capillary (CAP), interstitial (INT), and tumor cell. The former two are considered in the extracellular compartment (ESC). *k_RES_* represents reticuloendothelial system (RES) mediated elimination rate constant of liposomal doxorubicin; liposomal doxorubicin was unidirectionally transported from CAP into INT (*k*_tu_); *k*_rel_ represents the first-order release rate constant of free doxorubicin from liposomes in blood, CAP and INT; distribution of free doxorubicin to tumor cells was described using *k_te_* and *k*_et_; *k*_21_, *k*_12_, and *k*_10_ represent the micro-pharmacokinetic constants for free doxorubicin. For the pharmacodynamic model, a cell-kill kinetic model was linked with the PK model. The mass balance equation describes the change rate of cell number, where *C_s_* represents cell number, *f*_b_ *∙C*_ecs_ represents the unbound drug concentration in ESC, *k_s_* is the cell proliferation rate constant, and *k* is the drug-induced irreversible cell-death rate constant.

**Figure 8:**
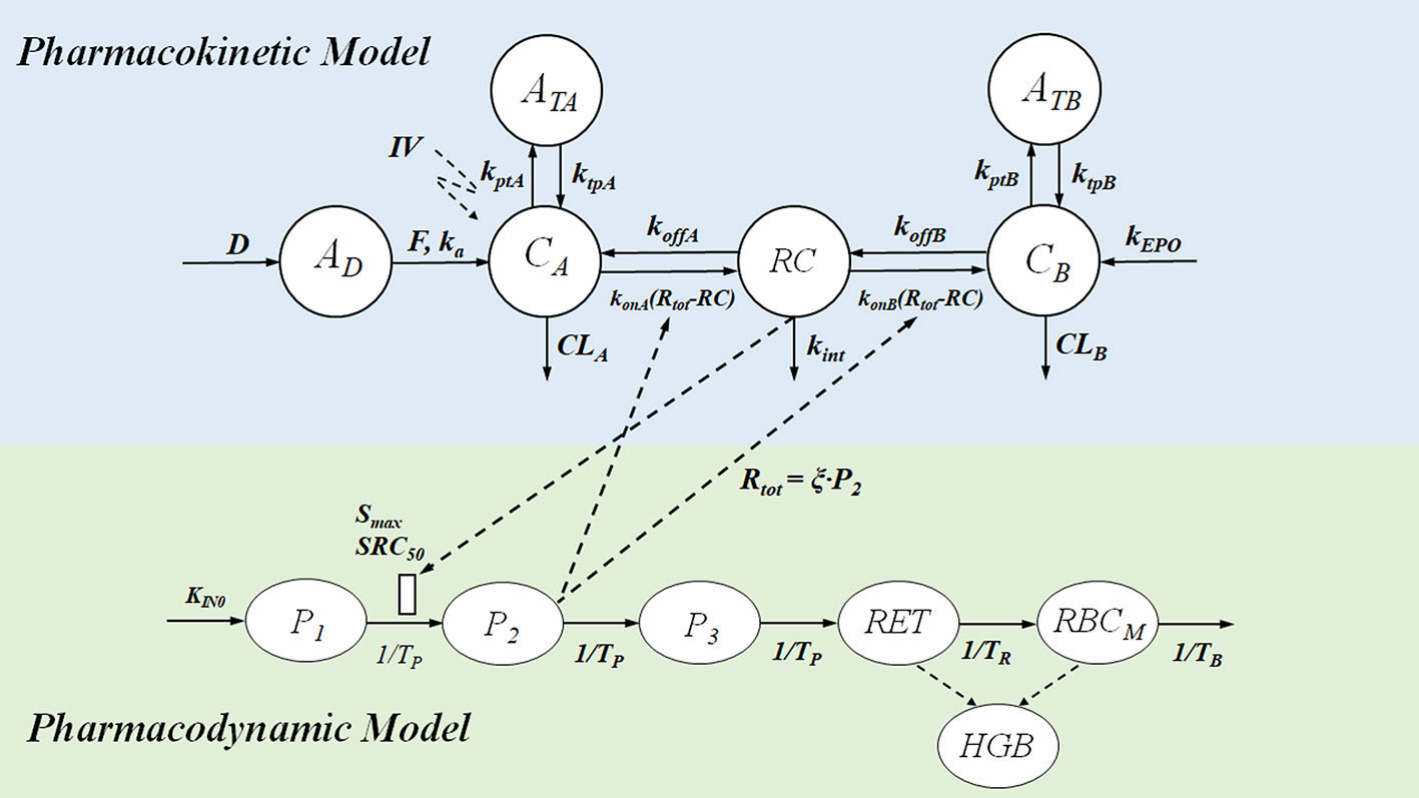
Mechanism-based PK-PD model of ESAs. The upper panel of the flow schematic is based on the TMDD model to describe the PK process. *D* and *A_D_* represent the duration of the zero-order input and absorption compartment for the SC route, respectively. *F* is the bioavailability, and *k_a_* is the first-order absorption rate constant. *C*_A_, *C*_B_, and RC represent the concentration of free ESAs, endogenous EPO (eEPO), and the EPO-receptor complex, respectively. A_TA_ and A_TB_ are tissue compartments, *k*_tpA_, *k*_tpB_, *k*_ptA_, and *k*_ptB_ are tissue distribution rate constants; *k*_onA_, *k*_onB_, *k*_offA_, and *k*_offB_ represents second-order rate constants for forming RC and first-order dissociation rate constants of ESAs and eEPO; *R_tot_* represents total receptor concentration; *CL_A_* and *CL_B_* are the first-order elimination rate constants; *k*_int_ is the first-order internalization and degradation rate constant; *k*_EPO_ represents the synthesis rate of eEPO. The PD model contains delay parameters, indicating the life span of various erythropoietic cells. *P*_1_, *P*_2_, and *P*_3_ represent three different maturation-level erythroid precursor cell compartments; *RET* is reticulocytes, and *RBCM* is mature red blood cell; HGB represents hemoglobin. *K*_IN0_ represents production process for *P_1_* cells, *S*_max_ represents the maximal effect of RC on the proliferation of precursor cells, and SRC_50_ is the concentration of drug-receptor complex to induce half of *S*_max_; *T*_P_, *T*_R_, *T*_B_, mean residence time for precursor cell, reticulocytes, and mature red blood cell. PD effect of the expansion of precursor cell on *R_tot_* mediates the PK process in return, and ξ is the factor of proportionality.

**Figure 9:**
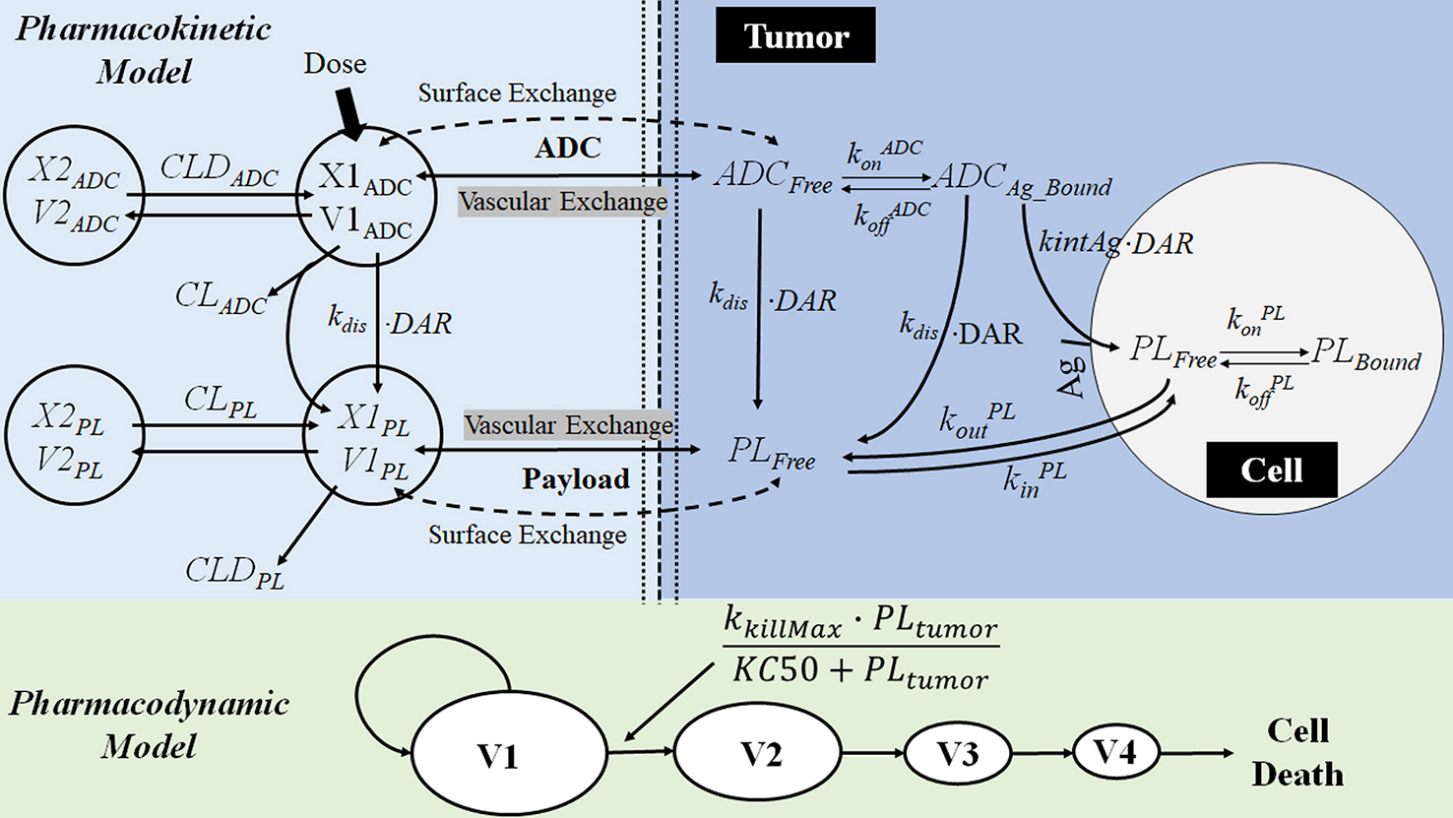
The multiscale PK-PD model of brentuximab-vedotin. The PK model is an integration of a modified two-compartment model in tumor to simultaneously characterize the plasma and intracellular PK of ADC and the payload. After the IV administration of ADC, partial ADC dissociates and releases payloads. ADC and the free drug in the central compartment can be eliminated, distribute to the peripheral compartment, or distribute to the tumor compartment. In the tumor tissue, the free drug could enter the cell by passive diffusion and bind to the target or go back into the extracellular environment. ADC in the tumor environment interacts with the antigen on the tumor cell membrane, and is internalized into the tumor cell. Subsequently, ADC is degraded in lysosomes to release the free drug intracellularly. The intracellular concentration is considered as the concentration in the biophase compartment. X1_ADC_, X2_ADC_, X1_PL_, and X2_PL_, the amount of ADC or payload in the central or peripheral compartment; V1_ADC_, V2_ADC_, V1_PL_, and V2_PL_, volume of distribution of ADC or payload in the central or peripheral compartment; CL_ADC_, plasma clearance of ADC; CLD_ADC_, distribution clearance of ADC; ADC_Free_ and ADC_Bound_, free and bound ADC concentration in tumor tissue; Ag: total antigen; PL_Free_ and PL_Bound_, free and bound payload concentration in cancer cell; DAR, average drug antibody ratio; *k*_dis_, dissociation rate of payload from antibody-drug conjugate; *k_i_*_nt_^Ag^, internalization rate of antigen inside the cell; *k*_on_^ADC^ and *k*_off_^ADC^, association and dissociation rate constants between antibody-drug conjugate and antigen; *k*_on_^PL^ and *k*_off_^PL^, association and dissociation rate constants between drug and intracellular drug target; *k_i_*_n_^PL^, drug nonspecific uptake rate in cancer cell; *k*_out_^PL^, efflux rate of payload from the cancer cell; V1, V2, V3, and V4, tumor volume in the growth.

**Table 1:** PK-PD model components and related mechanisms of action of drugs.

Drug	PK model components	PD model components	Contribution of PK-PD modeling
**Paliperidone palmitate**	One-compartment model with First-order elimination First-order absorption Zero-order absorption Flip-flop kinetics	Dopamine D_2_ receptor occupancy regulation (Kapur et al., 2000)	Optimized formulation and dose regimen Accelerated the clinical trial
**Liposomal doxorubicin**	Two-compartment model for doxorubicin One-compartment model for liposome Flip-flop kinetics	Biophase model (Sheiner et al., 1979) Cell-killing kinetic model (Jusko, 1973)	Evaluated the influence of drug-, carrier-, and system-specific parameter on anti-tumor efficacy
**Erythropoiesis stimulating agents**	Two-compartment model First-order absorption Flip-flop kinetics TMDD model (Mager and Jusko, 2001a)	The operational model of agonism (Black and Leff, 1983) Precursor-Pool dependent indirect response model (Sharma et al., 1998) Transit compartment model (Mager and Jusko, 2001b)	Quantified minimal-effect concentration Explained the relationship between *in vivo* binding affinity and effect
**Brentuximab-vedotin**	Two-compartment model with first-order elimination	E_max_ model (Wagner, 1968) Biophase model (Sheiner et al., 1979) Transit compartment model (Mager and Jusko, 2001b)	Predicted the clinical response by preclinical data

#### Application to Paliperidone Palmitate

The development of a 3-month extended-release (ER) formulation of paliperidone palmitate (PP3M), demonstrates how the PK-PD modeling can contribute to the development of new ER formulation. Paliperidone (9-hydroxy-risperidone) is an antipsychotic agent for the acute and maintenance treatment of schizophrenia (Samtani et al., 2009). PP3M is a long-acting injectable formulation of paliperidone palmitate, which is the insoluble ester prodrug of paliperidone (Cleton et al., 2014; Gopal et al., 2015). After intramuscular injection, paliperidone palmitate particles slowly dissolve into the local fluids at the injection site. Due to the poor aqueous solubility, the drug release from the formulation is a dissolution-driven process and becomes the rate-limiting step of absorption. Ultimately, the slow absorption resulted in flip-flop kinetics and prolonged apparent half-life.

The formulation of PP3M was developed from a 1-month formulation (PP1M) based on the knowledge gained from PK-PD modeling, which identified the injection volume as a significant factor affecting the release rate (Samtani et al., 2009). The PK-PD model of paliperidone palmitate is shown in **Figure 6**. For the PK modeling in PP1M, the deconvolution technique was utilized to identify the appropriate absorption model. A fraction of the dose was initially absorbed *via* a zero-order process, and the remaining fraction entered the central compartment *via* a first-order process after a delay. Since paliperidone is a dopamine receptor D_2_ antagonist, the clinical response is linked with the D_2_ receptor occupancy (Nordström et al., 1993; Kapur et al., 2000; Arakawa et al., 2008). The previous study has indicated that over 70% receptor occupancy is needed for the therapeutic effect (Arakawa et al., 2008). A PK model-based covariate analysis suggested the absorption rate (*k_a_*) of PP1M is negatively associated with injection volume. Thus, the PP3M formulation was developed with an increase in the injection volume. Together with the benefit of an increased drug concentration in the suspension, the half-life of the new formulation is long enough to maintain an effective concentration over three months (Samtani et al., 2009; Samtani et al., 2016).

PK-PD modeling also accelerated the clinical development of PP3M. Based on the Phase I study of PP3M, a population PK model was developed. Model-based simulations were conducted to find the dose with a PK profile matching that of the PP1M formulation in the Phase III study (Samtani et al., 2016). The PK matching strategy hypothesized that the drug effect was dependent on the drug concentration in plasma above a targeting concentration supported by the PP1M PK/PD studies. Eventually, based on the PK-PD study of PP1M and limited single-dose Phase I data of PP3M, a prospective dose in Phase III was selected without conducting any Phase II dose-finding study. The Phase III study in the end achieved predicted PK and efficacy. The development of the PP3M formulation is a successful case, demonstrating that PK-PD modeling can significantly accelerate the clinical development of a drug delivery system and reduce the cost.

#### Application to Long-Circulating Liposomal Doxorubicin

The PK-PD model in **Figure 7** described the disposition of liposomal doxorubicin and free doxorubicin, and quantitatively evaluated the relationship between drug exposure and anti-tumor effect by separating the carrier specific-, drug-specific- and system-specific parameters (Harashima et al., 1999). The model was developed based on reported and experimentally obtained preclinical data. The PK profile of liposomal doxorubicin and free doxorubicin in the blood were described by one- and two-compartment models, respectively. Extracellular (ESC) compartment at the tumor site is considered as the biophase compartment, where the concentration has been linked with a cell-killing function. A sensitivity analysis suggested that the rates of liposome clearance (*k*_RES_) and drug release (*k*_rel_) play critical roles in drug delivery. Lower *k*_RES_ could mitigate the loss of liposomal doxorubicin in blood to maintain a longer blood circulation period of liposomes. Subsequently, the accumulation of liposomes in the tumor site (ESC) would increase, resulting in a better tumor-killing effect. Furthermore, the simulation results suggested that a medium release rate (*k*_rel_ at 0.06 h^−1^) was optimal to achieve higher efficiency in rodents. Compared with the rapid-release formulations, a slower release rate could increase the free drug accumulation in the ESC compartment, instead of being cleared in blood. However, if the release rate was too low (*k_rel_* at 0.006 h^−1^), the formulation failed to achieve the critical drug concentration to inhibit tumor proliferation (Harashima et al., 1999). Therefore, the optimal medium release rate suggested by the model was a balance between the drug elimination from blood and the drug accumulation in the tumor site.

In this mechanism-based PK-PD model, the separation of the delivery-specific properties from the drug- and system-specific properties allows predicting the *in vivo* anti-tumor effect under different experimental conditions. One can predict the *in vivo* outcome in various tumor models by changing the system-specific parameter (e.g., the sensitivity of the tumor to the anti-cancer drug). Similarly, by simulating PD associated with various carrier-specific parameters (e.g., clearance of the carrier), one can compare the performance of different carriers. Hence, PK-PD modeling and simulation can predict the drug effect in different disease models for carriers with varying PK properties.

#### Application to Erythropoiesis-Stimulating Agents

Mechanism-based PK-PD modeling was applied to quantify the MEC of erythropoiesis-stimulating agents (ESAs), and explain the paradox that ESAs with lower binding affinity has a higher *in vivo* activity (Yan and Krzyzanski, 2013). By binding to the erythropoietin receptor (EPOR) on the membrane of erythroid precursor cells, ESAs stimulate the proliferation and differentiation of erythroid precursor cells (Elliott et al., 2008). Current ESAs include epoetin alfa, darbepoetin alfa, and continuous erythropoietin receptor activator (CERA) (Locatelli and Del Vecchio, 2011). Epoetin, the first recombinant human erythropoietin (rHuEPO), has a half-life between 5 and 12 hours and requires thrice-weekly dosing. Darbepoetin, a hyperglycosylated analog of rHuEPO, has 3- to 4-fold longer half-life than epoetin. However, the receptor binding affinity of darbepoetin is 4.3-times lower than that of epoetin, yet it has higher *in vivo* efficacy (Egrie and Browne, 2001). CERA was developed by incorporating a 30 kDa methoxy polyethylene glycol polymer chain to rHuEPO (Macdougall, 2005). It has a longer half-life, but its receptor binding affinity is much lower (50–100 times) than that of epoetin.

It was hypothesized that the effect of ESAs is not dependent on the peak concentration but on the duration of drug concentration above a ‘critical concentration’, also known as the MEC (Kiss et al., 2010). ESAs with a lower receptor-binding affinity are considered to have a higher MEC level to ensure sufficient receptor binding. Given MEC varies among various ESAs with different receptor binding affinity, the dosing of darbepoetin and CERA cannot be directly derived by PK matching with their predecessor, epoetin. Without the quantitative definition of MEC, a large number of clinical trials had been conducted to optimize the dosing regimen of darbepoetin (Glaspy et al., 2001; Glaspy et al., 2002; Vansteenkiste et al., 2002).

It was believed that establishing a relationship between the MEC and receptor binding affinity may help to find the optimal regimen for various ESAs. A mechanism-based PK-PD model was, therefore, developed to quantify the MEC of various ESAs based on the clinical PK-PD data of rHuEPO (Yan et al., 2012; Yan and Krzyzanski, 2013). The model structure is provided in **Figure 8**. This model incorporated the operational model of agonism into the PK-PD modeling, which helped to dissect the influence of receptor binding affinity on the drug effect. Furthermore, the binding between ESAs and EPOR results in receptor-mediated drug elimination (Yan and Krzyzanski, 2013). A model-based simulation was conducted to simulate the PK-PD profile of epoetin and darbepoetin under a thricely weekly IV bolus regimen. The PK profiles were overplayed with the C_50_ (Eq. 23) and showed that *C_50_* could be considered as the MEC:

(23) $$C_{50}=\frac{K_{D}}{1+\tau }$$

where *C_50_* is the ESA concentration that triggers the half-maximal effect, τ is the efficacy parameter in the operational model of agonism, and *K_D_* is the dissociation equilibrium constant of ESAs. Consistent with the MEC hypothesis, darbepoetin has with a lower binding affinity (higher *K_D_*), but a higher *C_50_* value compared to epoetin. However, the simulation also demonstrated that lower binding affinity of darbepoetin leads to lower receptor-mediated drug elimination and hence a slower clearance of the drug compared to epoetin. Therefore, the duration of darbepoetin concentration maintained above C_50_ is longer than that of epoetin, leading to a higher *in vivo* activity (Yan and Krzyzanski, 2013). In other words, the higher concentration and prolonged duration above MEC will eventually compensate for the counteracting effects of lower receptor binding affinity, thereby increasing their *in vivo* activities. Model-based simulations further successfully predicted that CERA had the highest *in vivo* potency among the three ESAs when administered with the same molar dose at any of the approved dosing regimens (i.e., thrice weekly, once weekly, and once every 2 weeks). The model also predicted that if the receptor binding affinity was too low, the benefit (i.e., longer half-life) of lower receptor binding affinity dissipated and eventually led to a lower *in vivo* activity.

#### Application to Brentuximab-Vedotin

PK-PD modeling is applied to improve the understanding of the complex PK-PD relationship of ADC. ADC consists of a monoclonal antibody (mAb), a cytotoxic payload, and a linker (Tsuchikama and An, 2018). After binding to the antigen expressed on the surface of a tumor cell, ADC is internalized and transported to lysosomes. Once inside of lysosomes, the linker is cleaved to release the cytotoxic payload. It can enhance the selective delivery of the payload to the tumor cell and reduce the systemic toxicity. Brentuximab-vedotin is an anti-CD30 based ADC (Shah et al., 2012). A multiscale PK-PD model (**Figure 9**) was developed to simultaneously captures the disposition of brentuximab-vedotin and its payload, at the cellular and physiological (plasma and tumor tissue) level. The detailed description of the model is provided in the legend of **Figure 9**. A cellular PK model was developed based on *in vitro* experiments, to mimic the intracellular and extracellular (tumor) PK activities of the payload and the ADC. Then, based on the preclinical and clinical *in vivo* data, the PK model of ADC and payload in plasma and tumor was developed. In terms of the PD model, the E_max_ model was used to describe the nonlinear relationship between the intracellular concentration and the tumor-killing rate. The PD parameters were estimated based on the preclinical tumor growth inhibition data in mice. By integration of the multiscale PK-PD model and PK data of brentuximab-vedotin in patients, clinical responses were predicted. A retrospective comparison suggested that model-predicted progression-free survival and complete response rates were comparable to those observed in clinical trials (Bartlett et al., 2009; Younes et al., 2010; Fanale et al., 2012). This example shows that the PK-PD model could integrate the *in vitro* and *in vivo* preclinical information to predict the ultimate clinical response.

Furthermore, this ADC disposition model offers a conceptual framework for the design of ADC and facilitates the preclinical-to-clinical translation. The PK model described above has been applied in auristatin-based anti-5T4 antibody-drug conjugates (Shah et al., 2014). It was discovered that the stability of the payload on the ADC and tumor size are the two most influential factors to the payload exposure in plasma. The linker controls the stability of payload and its dissociation from the antibody. The occurrence of extracellular dissociation increases the systemic exposure of toxic payload and results in a severe adverse effect. Therefore, it offered a rationale to modify the linker to increase the stability of the payload on ADC. Also, the sensitivity analysis suggested an increase in tumor size may lead to a rise in payload exposure in plasma. When tumors are small and avascular, ADC could only diffuse from the tumor periphery to the tumor. However, as a tumor grows and becomes vascularized, ADC can quickly enter the tumor by diffusion from the vasculature. Subsequently, the payload released from ADC inside of the tumor would also increase and diffuse to the systemic circulation. The author suggested that the difference in tumor size between animals and patients should be considered during the preclinical-to-clinical translation.

#### Model-Based Simulation to Illustrate the Hypothetical MEC

During the development of delivery system, maintaining drug concentration at a threshold concentration (e.g., IC_50_ and EC_50_) in blood or target site has often been used as a simple method to evaluate the performance of diverse formulation (Mishra et al., 2011). For example, drug release from nanocarriers is often deliberately controlled to maintain the plasma drug level within the therapeutic window between the MEC and the minimum toxic concentration (Siegel and Rathbone, 2012). The presented case studies also implied the presence of MEC and the importance of maintaining drug concentration above a MEC.

**Figure 10:**
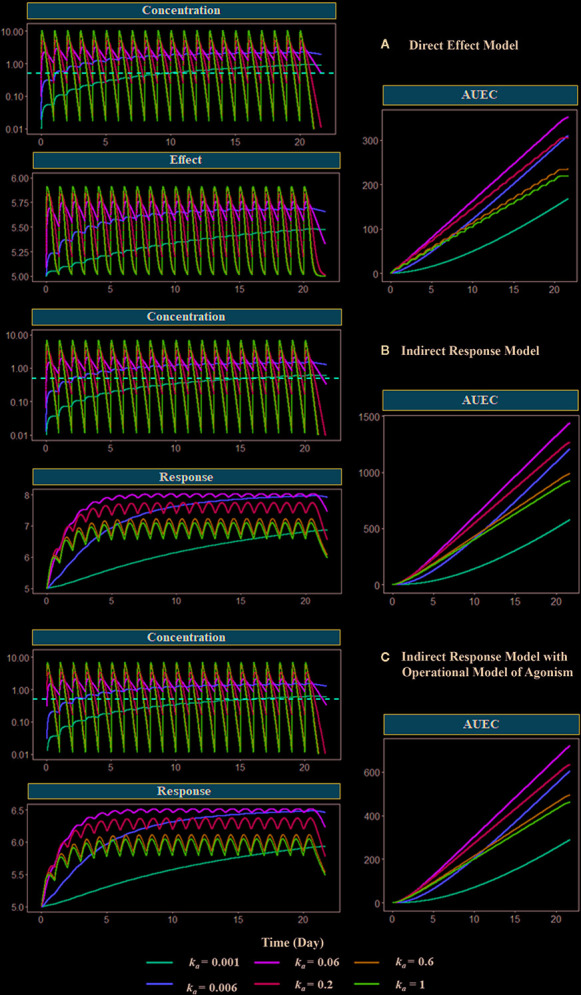
Model-based simulations with different absorption rates. Drug concentration, response (effect), and area under response (AUEC) versus time profiles have been simulated with different PD models: **(A)** direct response model, **(B)** indirect model, and **(C)** indirect response model with operational model of agonism. The dose is 1000 mg. The absorption rate varies from 0.001 to 1 h^−1^. The red dashed line shows the *EC_50_* values or the *A_50_* values, which are equal to 1 mg/L. Clearance (*CL*) is 27 L/h, the volume of distribution (*V*) is 90 L (*k_el_* = 0.3 h^−1^), concentration of receptor complex that triggers the half-maximum effect (*K*_E_) is 5 mg/L, E_max_ is 1, response production rate constant (*k*_in_) is 0.1 h^−1^, response elimination rate constant (*k*_out_) is 0.02 h^−1^, equilibrium dissociate rate (*K*_D_) is 2, target production (*k*_syn_) are 1 h^−1^, and degradation (*k*_deg_) rate are 0.2 h^−1^.

In this section, we use PK-PD modeling and simulation to illustrate the assertion and misconception of MEC. Simple direct response model (Eq. 12), indirect response model III (Eq. 20), and indirect model III with the operational model of agonism (Eqs. 15 and 20) are used in PD simulations to accommodate major mechanisms of drug action. A one-compartment model with first-order absorption and first-order elimination is used to simulate PK. The duration of drug concentration above a threshold was manipulated by changing the value of the absorption rate (*k_a_*), mimicking the different release rates of various drug delivery systems. EC_50_ and A_50_ are used as the concentration threshold in the simulation. The relationship between duration of drug concentration above a threshold, and the response was investigated. All the simulations were performed using mrgsolve, an open-source R package (Elmokadem et al., 2019). Parameter values are provided in the legend of **Figure 10**.

**Figure 10** shows the simulated PK, PD, and the area under the effect versus time curve (AUEC) for each model with different absorption rates (*k_a_*), which controls the duration of concentration above EC_50_ or A_50_. After the last dose treatment on day 21, the order of the AUEC under varying *k*_a_ values are *k*_a_ **= 0.06 > *k*_a_ = 0.2 > *k*_a_= 0.006 > *k*_a_ **= 0.6 > *k_a_*=1 > *k*_a_ = 0.001, which is consistent with the order of duration of drug concentration maintained above the threshold (red dashed line) in the PK profiles. The medium value of absorption *k*_a_ = 0.06 triggers the highest effect in all three models. Compared with *k*_a_ = 0.06 group, a larger *k*_a_ value (*k*_a_ = 0.2, 0.6, and 1) results in faster absorption and subsequent elimination, and a shorter duration of concentration above the threshold. On the other hand, when *k*_a_ is smaller (*k*_a_ = 0.006 and 0.001), the absorption is too slow to achieve the threshold concentration during the dosing period. These simulations demonstrate that the duration of drug concentration above a threshold is the determining factor for the therapeutic effect. Therefore, many formulations have focused on optimizing the drug release rate for maintaining drug concentration above MEC.

Although *EC*_50_ and *A*_50_ are used as the threshold in the present simulation, it should be noted that the threshold is a hypothetical concept and arbitrarily selected. The concentration-effect relationship (as expressed as E_max_ model or the operational model) is a continuous function without such a threshold. It means maintaining concentration above a specific threshold may not be appropriate for formulation selection, unless the concentration and response relationship is known, and the response associated with this threshold concentration is desired. Therefore, simulation based on the PK-PD relationship is preferred than a single threshold to select the formulation and dosing regimens.

## Challenge and Strategy

Although PK-PD modeling has facilitated drug development, several challenges are associated with its application. First, assumptions that are used to simplify the drug delivery process in PK-PD modeling might not always be appropriate. A reliable assumption requires a detailed understanding of both the physiological system and a drug delivery system. Due to the lack of relevant information, it’s challenging to validate these assumptions (Suryawanshi et al., 2010). For instance, the PK-PD model of ADC we discussed previously assumed the drug concentration in tumor cells was related to the cell-killing effect (Shah et al., 2012). However, without actual measurement in the distribution cascade, the validity of this assumption is uncertain.

Furthermore, extrapolation from animals to humans based on the PK-PD modeling is also challenging. For instance, in the case study of liposomal doxorubicin, the author suggested that the optimal rate of drug release in rodents was different from that in humans (Harashima et al., 1999). Although there is no universal solution, some methods could be considered to minimize the error of scaling. First, we should always select the appropriate animal species for scalling to humans (Tibbitts et al., 2010). For instance, it is known that dog is the most relevant species to human for preclinical cardiacsafety assessment (Gralinski, 2003; Dubois et al., 2017). Second, allometric scaling using multiple species has been applied to minimize the error and increase the accuracy of prediction (Huh et al., 2011). This method is commonly used to predict human PK (Amantana et al., 2013; Di Martino et al., 2019). Third, for some pharmacological systems, such as the erythropoietic system, the method of scaling from animal to human has already been established (Mager et al., 2009). Thus, given the available prior information, it is relatively more acceptable to scale from animal to human. Fourth, PK-PD modeling based on data collected from tumor xenograft mice model has been extensively used in oncology. This is supported by that the predicted threshold concentration derived from xenograft experiments correlates with the active dose in humans for several marketed chemotherapy drug (Rocchetti et al., 2007). Such a correlation can also be observed in targeted therapies, including both small and large molecules (Yamazaki, 2013; Lindauer et al., 2017). Therefore, the effective concentration in preclinical species could serve as a minimum target concentration that needs to be achieved in humans.

It should be pointed out that modeling assumptions are usually based on prior experience and knowledge and are not equal to random guessing (even they are often not be provided by modelers). They contribute to the power of modeling. Having assumptions helps us to fill the missing pieces among existing information and simplify the understanding of a complex system. However, modelers should be mindful of and acknowledge explicitly the assumptions and limitations of the model, and develop models that are fit for their purpose (Zhang et al., 2008). Methods like sensitivity analysis and external validation can check model’s dependency on assumptions, and allows to identify alternative assumption and the information that is needed to in future experiments. An optimal study design can be achieved by simulation and re-estimation, to ensure that the information collected in future experiments is informative on the new assumption (Suryawanshi et al., 2010). Once the new data become available to validate the predicted outcome, the assumption can be further refined and updated. Such a process can be repeated until the final goal of modeling is achieved.

## Summary

In this review, we have presented the basic theory and techniques of PK-PD modeling and highlighted its application in the development of different drug delivery systems and modified large molecules. PK-PD modeling and simulation are used to illustrate the misconception of the concentration threshold. The development of new technology can improve the understanding of the physicochemical properties of the delivery system and their interactions with the physiological system. Thus, the predictive ability of PK-PD modeling can be enhanced to guide the development of new drug delivery systems.

## Author Contributions

HZ performed the model simulation and wrote the manuscript. XY supervised and contributed to the simulation and editing of the review. SL and PB contributed to the revision of the review.

## Funding

This work was supported by the start‐up grant from School of Pharmacy, The Chinese University of Hong Kong.

## Conflict of Interest

The authors declare that the research was conducted in the absence of any commercial or financial relationships that could be construed as a potential conflict of interest.
